# Interconnections across physical, psychological, existential, and health-related quality of life patient-reported outcomes in adults with chronic medical conditions: Baseline findings from the eMPower randomized controlled trial

**DOI:** 10.1371/journal.pmen.0000694

**Published:** 2026-08-19

**Authors:** Emily Johnson, Ashley Hyde, Ben Vandermeer, Serena Isley, Shaina Corrick, Gail Wright, Justin Ezekowitz, Yinggan Zheng, Dayna Lee-Baggley, Holly Minckler, Jude Spiers, John C. Spence, Margaret L. McNeely, Stephanie Thompson, Kathleen Ismond, Sarah Tymchuk, Jennifer C. Lai, Puneeta Tandon

**Affiliations:** 1 Department of Medicine, Faculty of Medicine and Dentistry, University of Alberta, Edmonton, Alberta, Canada; 2 Faculty of Nursing, University of Alberta, Edmonton, Alberta, Canada; 3 Canadian PBC Society, Toronto, Ontario, Canada; 4 Department of Psychology, Saint Mary’s University, Halifax, Nova Scotia, Canada; 5 Faculty of Kinesiology, Sport, and Recreation, University of Alberta, Edmonton, Alberta, Canada; 6 Faculty of Rehabilitation Medicine, University of Alberta, Edmonton, Alberta, Canada; 7 Department of Psychiatry, University of Alberta, Edmonton, Alberta, Canada; 8 Department of Medicine, University of California, San Francisco, California, United States of America; Universiti Brunei Darussalam, BRUNEI DARUSSALAM

## Abstract

Individuals living with chronic illness commonly experience co-occurring physical, psychological, and existential symptoms that cluster and reinforce one another, contributing to functional decline and distress. Yet, frailty and existential distress have rarely been examined alongside psychological and physical symptoms, and few studies have mapped how these symptoms interrelate across chronic conditions. This study used baseline patient-reported outcome measures (PROMs) from the eMPower randomized controlled trial to examine interconnections among symptoms across physical, psychological, and existential domains in adults living with chronic medical conditions. Adults aged ≥18 years (n = 825) were enrolled in the 12-week eMPower digital mind-body program (breathwork/meditation practices, movement, psychology-based coping skills curriculum based on acceptance and commitment therapy (ACT)). Baseline PROMs included the Hospital Anxiety and Depression Scale (HADS), Modified Fatigue Impact Scale (MFIS), EQ-5D-5L, SF-12, Edmonton Frail Scale Acute Care version (EFS-AC), online Fried Frailty Phenotype (online FFP), Demoralization Scale-II (DS-II), and PROMIS Sleep Disturbance SF-8a. Network analysis of pooled baseline data using LASSO-regularized partial correlations identified clusters and central symptoms. Participants had a mean age 55.6 ± 12.7 years and were predominantly female (84%). Across the cohort, symptom burden was high, frailty was prevalent, and quality of life was impaired. Network analysis identified three clusters: (i) mental health; (ii) fatigue and frailty; and (iii) quality of life, with physical fatigue emerging as the most central node linking psychological and functional domains. These findings suggest that fatigue, demoralization, and frailty are interconnected drivers of symptom burden in chronic illness. Targeting fatigue may represent an important strategy for improving mental health and functional outcomes in people living with chronic medical conditions.

## Introduction

Individuals living with chronic illness commonly experience anxiety, depression, and fatigue [1], which can cluster and reinforce one another [2–6], contributing to functional decline, impaired health related quality of life [7], and distress [8]. These interdependencies highlight the need to examine symptoms not as isolated outcomes, but as interacting systems that can contribute to suffering [9,10]. Within this multidimensional framework, symptoms can be organized into three interconnected domains: physical (fatigue, frailty, mobility limitations), psychological (anxiety, depression), and existential and health-related quality of life (demoralization, health related quality of life) [11–13]. Of these symptoms, frailty and existential distress have been less commonly evaluated alongside psychological and physical symptoms in chronic disease research [14–16].

While these symptoms are common across chronic conditions, studies have most commonly investigated symptoms within single disease populations, such as cardiac disease [17], cancer [18] or multiple sclerosis [19], limiting cross-condition generalizability. Few studies have simultaneously assessed how physical, psychological, and existential symptoms correlate across chronic conditions, or what symptoms bridge these domains. Network analysis offers a method to visualize and quantify these relationships, identifying central symptoms that sustain distress and potential intervention targets [20]. Prior network studies in specific illnesses (cancer [21], multiple sclerosis [22], and post-acute sequelae of SARS-CoV-2 infection (PASC) [23]) have consistently identified fatigue as a central symptom, yet the broader structure linking fatigue, mood disturbance, frailty, and demoralization across chronic conditions remains uncharacterized. Characterizing this structure is particularly relevant for digital mind-body interventions, which aim to address multiple co-occurring symptoms simultaneously and therefore depend on understanding which symptoms are most central and interconnected.

The eMPower (evidence-based Mind-body Programming online for adults with chronic medical conditions) randomized controlled trial (RCT) was designed with this multidimensional symptom burden in mind. eMPower is a fully digital, 12-week integrative program grounded in acceptance and commitment therapy (ACT), a third-wave behavioural approach that targets psychological flexibility and values-based action rather than symptom elimination, making it well-suited to the existential and coping-related difficulties common in chronic illness [24]. Alongside the ACT-based coping skills curriculum, the program delivers condition-specific disease education, breathwork and meditation practices, and gentle movement (e.g., yoga, Tai-chi), with content co-developed with patient partners and clinical experts [25]. Mind-body and digitally delivered interventions of this kind have shown benefits for depression, anxiety, and fatigue across chronic physical conditions in prior systematic review evidence, though few have been evaluated across a broad, mixed chronic disease population [18]. The baseline data from 825 participants with diverse chronic conditions therefore provide a unique opportunity to map these multidimensional symptom relationships before intervention.

The specific objectives of this analysis are to: (i) characterize baseline symptom burden across physical (fatigue, frailty), psychological (anxiety, depression), and existential and health-related quality of life (demoralization, quality of life) domains; and (ii) identify central and bridging symptoms that may represent key targets for integrative interventions. To current knowledge, this is the first network analysis to integrate frailty and demoralization alongside psychological and physical symptoms in a large, mixed chronic disease cohort.

## Materials and methods

### Ethics statement

This study was approved by the University of Alberta Health Research Ethics Board – Health Panel (Pro00122568). All participants provided written informed consent electronically prior to study enrollment using Research Electronic Data Capture (REDCap) [26], and explicitly covered completion of online assessments and use of the digital intervention. The study was conducted in accordance with the principles of the Declaration of Helsinki and applicable institutional guidelines. Only adults aged 18 years or older were eligible to participate; therefore, parental or guardian consent for minors was not applicable.

### Design and analytic approach

This is a cross-sectional analysis of baseline assessments from the parent eMPower RCT [25]. The analysis used validated patient-reported outcome measures (PROMs) collected at enrollment to model symptom interrelationships across psychological, physical, and existential and health-related quality of life domains. The unit of analysis was individual symptoms, with participants serving as observational units. Edges in the network represent associations between symptoms. No clustering or classification of participants was performed. Analyses used pooled baseline data irrespective of future randomization arm, and no between-arm comparisons were undertaken.

### eMPower trial design and intervention

The eMPower trial is a three-armed, pragmatic RCT designed to evaluate a 12-week mind-body program for adults living with chronic medical conditions [27]. Participants were randomized to: (i) control, (ii) the 12-week program, or (iii) the 12-week program + weekly check-ins with study staff trained in motivational interviewing. Participants accessed online programs tailored to their condition (e.g., unique programs for cirrhosis, inflammatory bowel disease, heart failure, etc.). Programs included 90% shared core content and condition-specific educational videos co-developed with patient partners and clinical experts. The sample size for the trial was determined a priori to detect differences in the primary outcome between control and eMPower + weekly check-ins; details of assumptions and calculations are provided in the published protocol [25]. Reporting follows STROBE guidance for cross-sectional analyses, with parent-trial design and conduct reported per CONSORT in the protocol publication [28].

### Participants

Participants were aged ≥18 years, English speaking, had access to an internet connected device, and were primarily recruited from Canada and the United States, with additional participants joining internationally (see Table 1). Recruitment used multi-pronged outreach through patient partner organizations, clinic lists, social media, and the study website, with centralized online screening and electronic informed consent. All study procedures were conducted fully online, including enrollment and patient-reported outcome assessments administered using REDCap [26], and web-based intervention delivery. Participants self-identified as living with a chronic medical condition. Exclusion criteria included self-reported uncontrolled symptoms of schizophrenia, post-traumatic stress disorder, bipolar disorder, and/or suicidality nearly every day [25].

**Table 1:** Baseline characteristics of participants enrolled in the eMPower trial pooled across randomization arms.

	Total (n = 825)
**Age,** Mean (SD)	55.6 (12.7)
**Chronic medical condition,** n (%)	
“Other” chronic medical condition	254 (30.8%)
Primary Biliary Cholangitis (PBC)	184 (22.3%)
Digestive Disease(s)	128 (15.5%)
Post transplant	111 (13.5%)
Cirrhosis	51 (6.2%)
Heart failure	48 (5.8%)
Chronic kidney disease	29 (3.5%)
Primary sclerosing cholangitis (PSC)	20 (2.4%)
**Sex, Female**, n (%)	695 (84.2%)
**Gender, Woman,** n (%)	684 (82.9%)
**Work situation,** n (%)	
Retired or disabled/unable to work due to a disease	355 (43.0%)
Currently working	320 (38.8%)
On leave of absence	34 (4.1%)
Unemployed, looking for work	25 (3.0%)
Homemaker	18 (2.2%)
Student	15 (1.8%)
Volunteer	8 (1.0%)
Unemployed, not looking for work	9 (1.1%)
Other	41 (5.0%)
**Marital Status,** n (%)	
Married/Living common-law	554 (67.2%)
Single/Never married	124 (15.0%)
Divorced/Separated	120 (14.5%)
Widowed	27 (3.3%)
**Education level,** n (%)	
Completed post-secondary school (college/university)	578 (70.1%)
Some college/university	132 (16%)
Completed high school	61 (7.4%)
Completed registered apprenticeship/or other trades certificate	38 (4.6%)
No degree, certificate or diploma	16 (1.9%)
**Ethnicity,** n (%)	
White/Caucasian	730 (88.5%)
Southeast Asian	19 (2.3%)
Indigenous	17 (2.1%)
South Asian	13 (1.6%)
Black/African/Caribbean	12 (1.5%)
Latin American	11 (1.3%)
West Asian	2 (0.2%)
Arab	1 (0.1%)
Prefer not to answer	20 (2.4%)
**Region**, n (%)	
Urban	356 (43.2%)
Suburban	279 (33.8%)
Rural	183 (22.2%)
Remote	7 (0.8%)
**Baseline Technology Proficiency,** n (%)	
Not proficient/confident	5 (0.6%)
A little proficient/confident	18 (2.2%)
Somewhat proficient/confident	41 (5.0%)
Moderately proficient/confident	278 (33.7%)
Highly proficient/confident	329 (39.9%)
Technology “expert”	154 (18.7%)

### Baseline measures

Demographic variables included primary chronic medical health condition, age, sex, gender, ethnicity, and technological proficiency, measured using a scale from 0-5 (0 “Not proficient/confident with technology”, 5 “Technology ‘Expert’”). At enrollment, participants completed validated PROMs capturing symptoms across three interrelated domains: (i) physical, (ii) psychological, and (iii) existential and health-related quality of life. Unless otherwise specified, higher scores indicate greater symptom burden; exceptions (SF-12 PCS/MCS and the EQ-5D-5L visual analog scale, for which higher scores reflect better health) are noted below.

#### Physical domain.

Fatigue was assessed using the Modified Fatigue Impact Scale (MFIS [29]), a 21-item questionnaire scored from 0-84, comprising physical (0–36), cognitive (0–40), and psychosocial (0–8) subscales, with higher scores indicating greater fatigue impact. Frailty was assessed using two instruments. The Edmonton Frail Scale Acute Care version (EFS-AC) [30] ranges from 0 to 17, with higher scores indicating greater frailty; participants were categorized as fit (0–3), vulnerable (4 –5), or mildly (6 –7), moderately (8 –9), or severely (≥10) frail. The online Fried Frailty Phenotype (online FFP) [31], an adapted version of the original Fried Frailty Phenotype, classifies participants on five self-reported physical criteria (unintentional weight loss, decreased grip strength, increased fatigue or declining endurance, slowed walking pace, and decline in activity level); participants meeting 0 criteria were classified as non-frail, 1–2 as pre-frail, and ≥3 as frail. Sleep disturbance was evaluated using the PROMIS Sleep Disturbance Short Form – 8a (SF-8a) [32], reported as a T-score normalized to a mean of 50 and standard deviation of 10 in the reference population, with higher scores indicating greater sleep disturbance.

#### Psychological domain.

Anxiety and depression were measured with the Hospital Anxiety and Depression Scale (HADS), comprising anxiety (HADS-A) and depression (HADS-D) subscales [33] of seven items each. Each subscale ranges from 0-21 (total 0–42), with higher scores indicating greater symptom burden.

#### Existential and health-related quality of life domain.

Demoralization is characterized by helplessness, hopelessness, and loss of purpose. It captures an existential dimension distinct from depression and has been linked to worse mental health and lower quality of life [34]. Demoralization was measured using the 16-item Demoralization-II scale (DS-II) [35], scored from 0-32, comprising meaning/purpose and distress/coping subscales (each 0–16), with higher scores indicating greater demoralization. Total scores were categorized as low (0–3), moderate (4 –10), or high (≥11) demoralization [35]. Health-related quality of life was evaluated using the EQ-5D-5L [36] and the Short Form-12 (SF-12) [37]. The EQ-5D-5L comprises five dimensions (mobility, self-care, usual activities, pain/discomfort, and anxiety/depression), each rated from 1 (no problems) to 5 (extreme problems), summed to a total ranging from 5-25 where higher scores indicate greater problems, accompanied by a visual analog scale (VAS) or self-rated health from 0-100 where higher scores indicate better health. The SF-12 yields physical (PCS) and mental component summary (MCS) scores normalized to a mean of 50 and standard deviation of 10 in the general population, with higher scores reflecting better health status. Although the SF-12 PCS and MCS include physical and psychological components, these measures were categorized within the health-related quality of life domain because they reflect broader perceptions of overall functioning and health status rather than isolated symptom constructs.

### Network analytic approach

Analyses proceeded in two stages to examine how symptoms across physical, psychological, and existential and health-related quality of life domains interrelated at baseline. First, pairwise Spearman correlations were computed among all PROMs to quantify direct associations within and between domains. Second, a LASSO regularized partial correlation was estimated using the qgraph package in R (Version 4.5.2). This approach identifies meaningful connections between PROMs while controlling for all others, minimizing spurious associations [38,39]. In the resulting network, nodes represented PROMs (subscales where available or total scales), and edges represented partial correlations that quantify the strength and direction of inter-symptom relationships. The primary network included all PROMs across the three symptom domains to allow assessment of both within-domain clustering and cross-domain bridging patterns. This baseline network analysis was exploratory and hypothesis-generating; no a priori edge or centrality-specific hypotheses were pre-specified. To examine robustness, a sensitivity analyses incorporated age as a continuous variable using identical model specifications and visualization to assess the consistency of community structure and node-strength rankings.

Networks were visualized using the Fruchterman-Reingold algorithm, where blue edges represent positive associations, red edges negative associations, and edge thickness represents magnitude. Community detection used the Spinglass algorithm to identify densely connected clusters of symptoms [40]. Node centrality was quantified using expected influence (EI1 and EI2), which captures both direct and indirect connections. Bridge centrality identified symptoms linking different domains, highlighting potential cross-domain targets for intervention. Network stability was evaluated through nonparametric bootstrapping (1000 samples) and case-dropping procedures, with a target correlation stability (CS) coefficient ≥0.7. Missing data were handled using pairwise deletion to maximize available observations per edge, appropriate when missingness is approximately missing completely at random/missing at random and commonly used in network estimation; robustness was checked with bootstrapping and subgroup analyses [38]. HADS-A, HADS-D, MFIS, EQ-5D-5L, EFS-AC, SF-12 scores were complete for all participants, whereas DS-II, online FFP, and Sleep Disturbance SF-8 had partial missingness (9% for DS-II and online FFP; 19% for Sleep disturbance SF-8a). The SF-12 PCS was excluded because its scoring renders it uncorrelated with the MCS [41]. Both partial correlations (from the network) and pairwise correlations (direct associations between two symptoms without controlling for other symptoms) were examined to characterize relationships among symptom domains.

### Baseline descriptive analyses

Descriptive data are presented as counts (%) for categorical variables and means ± standard deviations (SD) for continuous variables. Participant characteristics are summarized overall. Analyses were conducted in SPSS, version 24 [42].

## Results

### Enrollment of eMPower study participants

Of 1,620 participants who initiated enrollment, 825 were randomized. Among the 795 participants not randomized, the primary reasons for non-enrollment included incomplete baseline surveys (n = 363; 45.7%), failure to attend a study orientation session (n = 229; 28.8%), and uncontrolled psychiatric symptoms identified during screening (n = 104; 13.1%). Remaining exclusions were due to miscellaneous reasons.

### Baseline characteristics

The first participant was randomized February 12, 2023, and enrollment concluded June 16, 2024, with final 12-week outcomes collected in December 2024. Baseline characteristics of randomized participants are summarized in Table 1. The mean age was 55.6 ± 12.7 years, with 24% of participants aged >65 years. Most participants were female (84%), and identified as white (88.5%), and were from North America (96.8%). Common primary chronic conditions included primary biliary cholangitis (PBC) (22.3%), chronic digestive disease (15.5%), post-transplant (13.5%), and another type of chronic medical condition not on the baseline questionnaire (“other”) (30.8%). For “other”, most conditions fell under cardiovascular (e.g., cardiovascular disease, hypertension), rheumatologic, musculoskeletal, or neurologic categories.

Self-reported technology proficiency was moderate to high for most participants (34% moderate, 40% high). Nearly half were retired or unable to work (43%), and 49% were the primary earners. Most (73%) reported being the primary person responsible for housework and lived with an average of 1.8 (± 1.3) people. Home stress was moderate (mean 4.9 ± 2.3 out of 10), and most participants were partnered (67%) and had completed post-secondary education (70%).

### Baseline symptoms

#### Physical domain.

Participants demonstrated high physical and functional symptom burden (Table 2). Fatigue was prominent, with a mean MFIS score of 45.4 ± 16.0, indicating moderate to severe fatigue. Subdomain scores were also elevated: physical (21.8 ± 7.8), cognitive (19.3 ± 8.1), and psychosocial (4.3 ± 2.0).

**Table 2:** Baseline PROMs across domains in the eMPower trial (n = 825, pooled across randomization arms).

Patient Reported Outcome Measure	Total (n = 825)
**HADS Total, mean (SD)**	13.5 (6.5)
HADS-Anxiety	8.0 (4.0)
HADS-Depression	5.5 (3.5)
**MFIS Total, mean (SD)**	45.4 (16.0)
MFIS Physical	21.8 (7.8)
MFIS Cognitive	19.3 (8.1)
MFIS Psychosocial	4.3 (2.0)
**EQ-5D-5L Total, mean (SD)**	9.9 (3.3)
*EQ-5D-5L Mobility*	
No problems n (%)	421 (51.0%)
Slight problems	207 (25.1%)
Moderate problems	146 (17.7%)
Severe problems	48 (5.8%)
Extreme problems	3 (0.4%)
*EQ-5D-5L Self Care*	
No problems, n (%)	664 (80.5%)
Slight problems	99 (12.0%)
Moderate problems	53 (6.4%)
Severe problems	7 (0.8%)
Extreme problems	2 (0.3%)
*EQ-5D-5L Usual Activities*	
No problems, n (%)	249 (30.2%)
Slight problems	298 (36.1%)
Moderate problems	195 (23.6%)
Severe problems	67 (8.2%)
Extreme problems	16 (1.9%)
*EQ-5D-5L Pain*	
No problems, n (%)	109 (13.2%)
Slight problems	331 (40.2%)
Moderate problems	280 (33.9%)
Severe problems	87 (10.5%)
Extreme problems	18 (2.2%)
*EQ-5D-5L Anxiety/Depression*	
No problems, n (%)	185 (22.4%)
Slight problems	338 (41.0%)
Moderate problems	259 (31.4%)
Severe problems	39 (4.7%)
Extreme problems	4 (0.5%)
*EQ-5D-5L VAS*, mean (SD)	61.6 (18.8)
**Quality of Life (SF-12),** mean (SD)	
Physical Component Summary (PCS) score	39.2 (10.6)
Mental Component Summary (MCS) score	42.2 (10.3)
**EFS-AC Total**, mean (SD)	5.0 (2.7)
*EFS-AC Frailty Status, n (%)*	
Fit	256 (31.0%)
Vulnerable	239 (29.0%)
Mild frailty	186 (22.5%)
Moderate frailty	91(11.0%)
Severe frailty	53 (6.4%)
**Online FFP Total (n = 751),** mean (SD)	2.6 (1.5)
*Online FFP Frailty Status, n (%)*	
Non-frail	92 (12.3%)
Pre-frail	238 (31.7%)
Frail	420 (56.1%)
**DS-II, Total (n = 751),** mean (SD)	10.1 (6.6)
Meaning/Purpose subscale	3.7 (3.5)
Distress/Coping subscale	6.4 (3.6)
DS-II Demoralization Status, n (%)	
Low (0–3)	134 (17.8%)
Moderate (4 –10)	287 (38.2%)
High (≥11)	330 (43.9%)
**Sleep Disturbance SF-8a, Total (n = 665)**	53.5 (3.8)

VAS=Visual Analog Scale; Online FFP = online Fried Frailty Phenotype; DS-II = Demoralization-II Scale.

Frailty was common, with 56.1% classified as “Frail” and an additional 31.7% “Pre-frail” according to the online FFP. Similarly, on the EFS-AC, > 15% of participants scored within the “Moderate” or “Severe” frailty categories.

Sleep disturbance was elevated, with mean SF-8a scores of 53.5 ± 3.8, consistent with significant sleep disruption [32]. Pain was also frequent, with mean EQ-5D-5L pain scores of 2.5 ± 0.9, and over two-thirds of participants reported some level of pain, including >10% rating it as severe or extreme.

#### Psychological domain.

Mean HADS-A scores (8.0 ± 4.0) were higher than mean HADS-D scores (5.5 ± 3.5). Only 22% reported no anxiety or depression symptoms, while more than one-third reported moderate to extreme symptoms on the EQ-5D-5L mental health question.

#### Existential and health-related quality of life domain.

Demoralization was elevated (10.1 ± 6.6), particularly within the distress/coping ability subscale (6.4 ± 3.6), with 43.9% meeting criteria for high demoralization [35].

Quality of life was impaired across global and health-related measures. SF-12 MCS scores averaged 42.2 ± 10.3, and PCS 39.2 ± 10.6, both below population norms [43]. The mean EQ-5D-5L VAS was 61.6 ± 18.8, underscoring diminished perceived health status.

### Symptom correlations

Pairwise correlations demonstrated strong interconnections within and across domains. Within the psychological and existential clusters, anxiety, depression, and demoralization were strongly correlated (r = 0.50—0.62). Correlations were observed between fatigue subdomains (physical, cognitive, psychosocial) and depression (r = 0.46—0.58). Frailty measures correlated moderately with pain and mobility (r ~ 0.40).

### Network analysis

**Fig 1:**
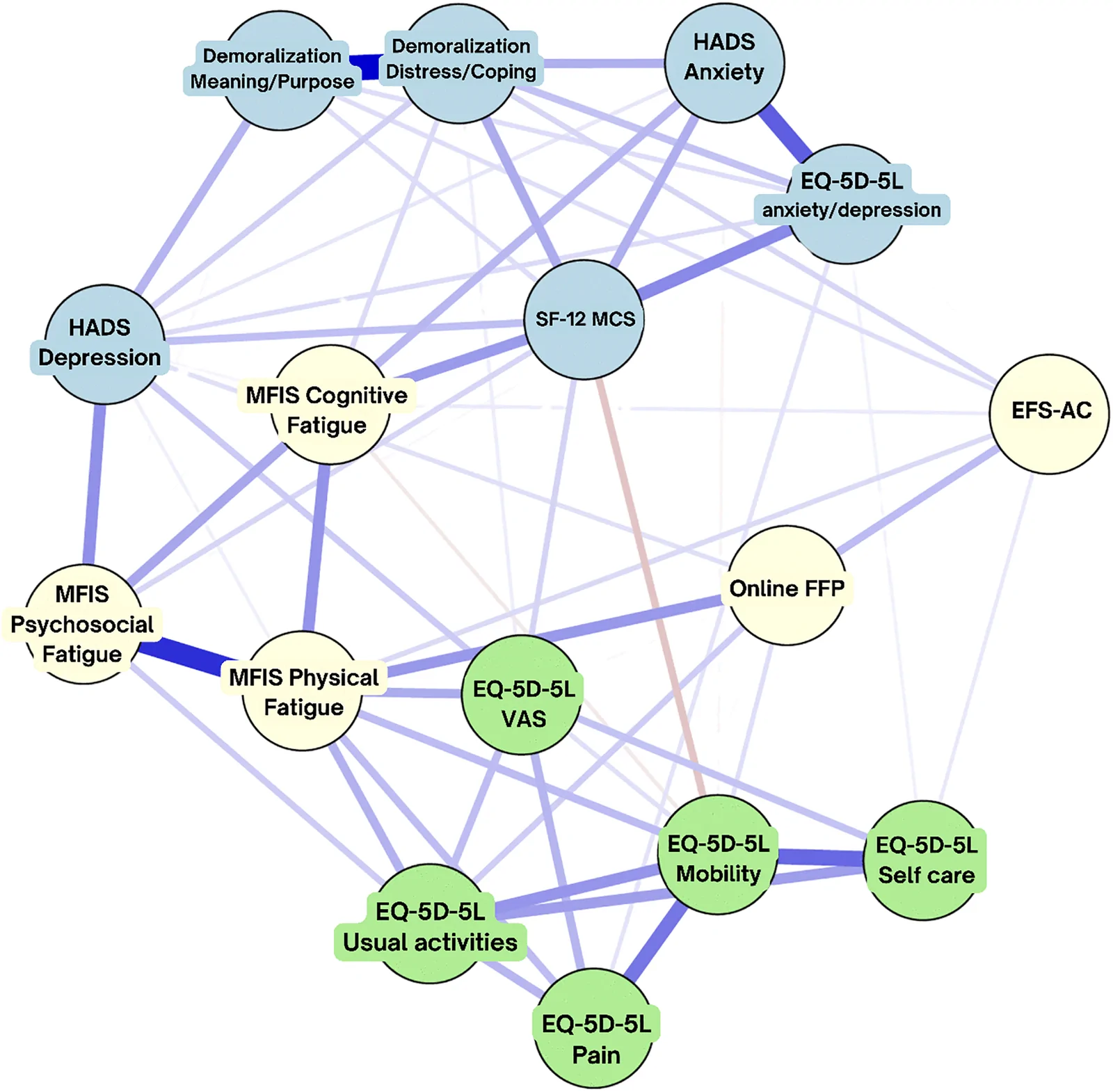
Network analysis. Network visualization of regularized partial correlations among baseline patient-reported outcome measures (PROMs). Nodes represent symptom domains or PROM subscales, and edges represent unique associations between symptoms after controlling for all other variables in the network. Blue edges indicate positive associations, and red edges indicate negative associations, with thicker edges representing stronger associations. Node colours reflect symptom groupings identified through community detection analysis: psychological and existential symptoms (blue), fatigue and frailty symptoms (beige), and health-related quality of life measures (green). HADS = Hospital Anxiety and Depression Scale; MFIS = Modified Fatigue Impact Scale; EQ-5D-5L = EuroQol 5-Dimension 5-Level; SF-12 MCS = Short Form-12 Mental Component Summary; EFS-AC = Edmonton Frail Scale Acute Care version; Online FFP = online Fried Frailty Phenotype.

The partial correlation network (Fig 1) revealed three primary symptom clusters: (i) mental health; (ii) fatigue and frailty; and (iii) existential and health-related quality of life. Physical fatigue had the highest node strength (1.52), followed by demoralization distress/coping (1.20), EQ-5D-5L mobility (1.20), and SF-12 MCS (1.20).

To assess the robustness of these findings, an exploratory network was estimated with age as a continuous node. This network revealed an expected frailty–age association but did not alter the overall network structure (S1 Fig). These data-driven clusters partially overlapped with, but were not identical to, the conceptual symptom domains used to categorize PROMs in the Methods section.

## Discussion

In this baseline network analysis of 825 participants in the eMPower RCT, symptoms across physical, psychological and existential and health-related quality of life domains demonstrated substantial interconnectivity, with fatigue, frailty, and mental health symptoms showing strong associations. These findings are consistent with prior network analyses that have mapped associations among psychological and somatic symptoms in aging and population-based cohorts [9,44], including work linking depressive symptoms, sleep, and frailty in older adults, and transdiagnostic networks of internalizing and functional symptoms in the general population. However, those studies primarily involved older or general-population cohorts rather than adults living with diverse chronic conditions, as examined in eMPower. Although network models are primarily hypothesis-generating, they yield valuable insight into the structure and interdependence of symptoms, informing refinement of intervention targets rather than prescribing them. The present analysis extends this literature by applying network methods to baseline PROMs from a large, cross-condition digital mind-body trial. By integrating physical, psychological, and existential and health-related quality of life domains, including fatigue, frailty, and demoralization, it advances understanding of how overlapping symptom systems interact in individuals with chronic illness.

Fatigue was moderate to severe across all domains and was closely linked to mental and psychosocial functioning [45]. Prior studies across chronic fatigue syndrome [45], chronic liver disease [46], inflammatory bowel disease [47], and cardiovascular disease [48] have documented strong links between fatigue and cognitive impairment. The growing recognition of cognitive fatigue or “brain fog” as a contributor to disability [49] underscores the importance of addressing fatigue’s full spectrum in chronic conditions [49,50]. Reviews of non-pharmacological trials suggest that mind-body strategies, including cognitive behavioural therapy (CBT) and cognitive pacing tools, can reduce fatigue severity and improve functional outcomes [51,52]. These patterns mirror trends observed in large-scale population cohorts. In the UK Biobank, over one-third of adults reported persistent fatigue, which was strongly associated with multimorbidity, psychological distress, sleep disturbance, and reduced physical activity, particularly among women and people 40–65 years of age [53,54]. Similarly, in the All of Us Research Program—a large, diverse U.S. population cohort—37.2% of participants reported moderate to severe fatigue, with fatigue more common among those with multiple chronic conditions, and associated with lower quality of life, physical limitations, and psychological distress [55]. Consistent with prior network analyses in general population and clinical cohorts (n = 300–1,000), fatigue, concentration difficulties, and sleep disturbance act as bridge symptoms linking psychological and physical domains [9,56,57]. Most of these studies examined psychiatric or post-infection populations and focused primarily on psychological or functional symptoms, without incorporating broader multidimensional constructs such as frailty or demoralization.

Although fatigue’s prominence could reflect its high prevalence across chronic illness, its strong connectivity with both psychological and physical symptoms in this network suggests broader mechanistic importance. Fatigue was not only common but also highly interlinked with anxiety, depression, demoralization, and mobility, indicating potential cross-domain influence. Targeting fatigue may therefore represent a promising strategy for improving multiple related symptoms, supporting its interpretation as a central and modifiable treatment target rather than a nonspecific manifestation of disease.

Over 55% of participants met self-reported physical frailty criteria, and an additional 31.7% met criteria for “pre-frail” according to the online FFP [31]. Network analysis linked physical frailty with mobility and pain, reinforcing its role as a marker of multimorbidity rather than age alone [58]. A distinct frailty-age cluster revealed that older adults reported more physical limitations, while younger adults reported greater emotional and cognitive distress. This aligns with population-level data showing increasing frailty among men and women over 35 years of age, driven by social and behavioral factors including diet, physical inactivity, smoking, and alcohol consumption [59]. These findings underscore the need for innovative, scalable interventions that are not only accessible but also tailored to the evolving needs of a diverse, younger demographic. In particular, online integrative interventions that combine physical activity, psychological support, and flexible delivery formats may offer greater relevance and acceptability for this population [60].

Anxiety emerged as a more prominent driver of psychological distress than depression. High demoralization scores indicated that distress extended beyond emotions to existential concerns, such as difficulty coping with chronic illness and a reduced sense of meaning or purpose. This aligns with qualitative research describing “illness-related identity disruption,” a shift in self-perception that negatively impacts adherence and health outcomes [61]. Stronger illness identity has been associated with reduced self-management, including reduced medication adherence, increased hospitalization, and worsened biomarkers [62]. Despite this, most digital mind-body interventions have not explicitly targeted demoralization or existential concerns. Future research could incorporate identity and meaning-focused strategies from CBT and ACT.

### Strengths and limitations

Strengths of this study include the large sample size, inclusion of diverse chronic medical health conditions, broad recruitment, and comprehensive patient-reported symptom assessment. Limitations include the extensive baseline surveys (~50 minutes) that may have introduced response fatigue, potentially adding variability in symptom reporting. As in other digital symptom management trials, males and non-white participants were underrepresented, limiting generalizability. Because participation required internet access and digital literacy, selection bias toward more digitally proficient participants was also possible. A further limitation is the absence of medical verification of participants’ conditions. Although self-report was a pragmatic necessity given the fully remote, multi-condition, and international recruitment design, it may have introduced misclassification of the primary condition and cannot exclude the possibility that some participants did not meet formal diagnostic criteria. Findings should therefore be interpreted as reflecting broad cross-condition symptom patterns rather than diagnosis-specific estimates. Future research should seek to incorporate objective clinical verification, such as linkage to medical records or clinician-confirmed diagnoses, where feasible. Representativeness was further influenced by higher enrolment of specific conditions (e.g., PBC), reflecting established patient partner networks; thus, findings reflect cross-condition patterns rather than population estimates by condition. Furthermore, the symptom network analysis is cross-sectional and correlational by design and cannot infer directionality or causality between symptoms. While bootstrapping supported stability, findings should be interpreted as hypothesis-generating rather than confirmatory. In addition, network analytic methods have intrinsic limitations. The structure and centrality estimates depend on the measures included, the regularization parameters, and sample characteristics. Highly correlated constructs may inflate node strength or bridge centrality, and networks estimated from cross-sectional data may differ from those based on temporal or longitudinal information. Future longitudinal or dynamic network analyses could clarify symptom interdependencies over time.

## Conclusions

In this secondary network analysis of baseline PROMs from a large and diverse cohort of adults with chronic medical conditions, strong interconnections were observed among physical, psychological, and existential domains. Fatigue emerged as a central symptom, bridging mental and physical health processes. These findings highlight the multidimensional nature of the symptom burden in chronic illness and suggest that targeting central symptoms – particularly fatigue – may produce cross domain benefits in function and mental wellbeing. This analysis demonstrates the utility of network methods for providing a framework for identifying modifiable targets, and informing the design of integrated health interventions for individuals living with chronic illness.

## Supporting information

S1 Fig Network analysis for all participants with age. Footnote: Exploratory symptom network including age as a continuous variable. The overall structure remained consistent with the primary network, but a distinct frailty–age cluster emerged, linking higher age with greater frailty and mobility limitations. This sensitivity analysis confirms the robustness of the primary network structure and highlights age-related variation in physical health domains. HADS = Hospital Anxiety and Depression Scale; EFS-AC = Edmonton Frail Scale Acute Care version; online FFP = online Fried Frailty Phenotype; MFIS = Modified Fatigue Impact Scale; EQ-5D-5L = EuroQol 5-Dimension 5-Level; SF-12 = Short Form-12; MCS = Mental Component Summary.
(TIF)
